# Nitrous Oxide Gas in Capital Operations

**Published:** 1866-02

**Authors:** J. M. Carnochan

**Affiliations:** Medical and Surgical Reporter


### NITROUS-OXIDE GAS IN CAPITAL OPERATIONS.

I observe in your last number, January 27th, a communication from Dr. Ellsworth, of Hartford, in which he states that I was in error in supposing that I was the first to perform a capital surgical operation under the influence of nitrous-oxide gas, as he had amputated a limb eighteen years ago, Dr. Wells himself administering the gas. I was aware, when I wrote my letter on this subject, that one or more surgical operations had been performed in Hartford, about the time of Wells' discovery. I therefore, if you will observe, distinctly stated that mine are the first capital operations performed under the influence of the gas, *since* the discovery of Wells, meaning since that epoch. I did not lay claim to any priority in the use of the gas, nor to any particular merit, except that of reviving its use for surgical purposes, after it had been abandoned in favor of chloroform and ether for nearly eighteen years, and proving and corroborating the fact that it is preferable to either, and perfectly suitable for all surgical operations of short duration.

I am pleased to find that so distinguished a surgeon as Dr. Ellsworth, should have been one of the first to practically demonstrate the anæsthetic qualities of nitrous-oxide gas in surgery proper.

Since my letter in December, I have performed four more capital operations upon adults, viz., one amputation of the thigh, one of the leg, the removal of a tumor from the side, and the extraction of a cataract, making in all, since last July, seven successful capital operations under the influence of anæsthesia produced by the nitrous-oxide gas. I have also, during this time, used chloroform and ether in my operations, and my opinion in regard to the superiority of the nitrous-oxide as an anæsthetic, is still unchanged. I believe, however, that there is a great room for improvement in the mode of administration of the gas; one principal fault at present being the repeated inhalation of the same material. An instrument which will act by a valvular arrangement, as in Reed's stomach-pump, would obviate this difficulty, and I have no doubt but that some skillful mechanician will produce one that will meet the necessary requirements.

The necessity which exists for some anæsthetic agent which will enable the patient to place himself in the hands of the operator without fear of the unpleasant, dangerous, and sometimes fatal effects of chloroform and sulphuric ether, renders the consideration of this subject a matter of much importance to the profession and to the world at large, and the elucidation and record of new facts connected with it may be made the basis of future improvements.

With the exception of the undoubted claim which WELLS has to the discovery of anæsthesia for surgical purposes, I regard the historical part of the subject as of comparatively small import. As the matter now stands, however, by reference to Senator TRUMAN SMITH'S Congressional report, referred to by Dr. ELLSWORTH, I find that on August 17th, 1847, Dr. E. E. MERCY, then of Hartford, now of this city, removed a schirrous testicle; that on January 1st, 1848, Dr. P. W. ELLSWORTH, of Hartford, performed an amputation of the thigh on a boy, and that, three days after, Dr. S. B.

BERESFORD, of the same place, removed an adipose tumor, six ounces in weight, from the shoulder of an adult.

Since these operations, performed at the time of WELLS' discovery, nitrous-oxide gas, as an anæsthetic, has been absolutely abandoned in capital surgery, until my operation, the extirpation of a cancerous mammary tumor, with enlarged cancerous glands in the axilla, on the 22d of July, 1865, an interval of about eighteen years. The period of time (sixteen minutes) that the patient was kept under the influence of the gas, during that operation, is, as far as I know, the longest on record. I must state, however, that during this time, the bag containing the gas was removed several times from the mouth of the patient, in order that the lungs might be injected with atmospheric air.

The subject remains open for future experiment to develop the further capabilities of the nitrous oxide gas as an anæsthetic agent.

J. M. CARNOCHAN, M. D.

*Medical and Surgical Reporter.*

---

